# Indications, Efficacy, and Complications of Pediatric Bronchoscopy: A Retrospective Study at a Tertiary Center

**DOI:** 10.7759/cureus.40888

**Published:** 2023-06-24

**Authors:** Sinan Yavuz, Amal Sherif, Safiya Saif, Asma Alzamar, Doaa Alawad, Ahmed Abdelwahab, Maged N Nabawi, Maryam Amirrad, Nader Francis

**Affiliations:** 1 Pediatrics/Pediatric Pulmonology, Al Qassimi Woman's and Children's Hospital, Sharjah, ARE; 2 Pediatrics, Al Qassimi Woman's and Children's Hospital, Sharjah, ARE; 3 Pediatrics/Pediatric Intensive Care, Al Qassimi Woman's and Children's Hospital, Sharjah, ARE; 4 Education, Uinversity of Ottawa, Ottawa, CAN; 5 Pediatrics, Royal College of Pediatrics & Child Health, London, GBR; 6 Pediatrics, Ajman University, Ajman, ARE

**Keywords:** cough, tracheomalacia, foreign body aspiration, bronchoscopy, rigid and fiber-optic bronchoscopy, flexible bronchoscopy, pediatric pneumonology

## Abstract

Background

Bronchoscopy is an essential procedure for evaluating, diagnosing, and treating pediatric respiratory diseases. In this study, we demonstrate the indications and contraindications of bronchoscopy done in a tertiary referral hospital, Al Qassimi Woman's and Children's Hospital (AQWCH) in Sharjah, United Arab Emirates (UAE), in order to achieve better service. This study aims to evaluate patients' characteristics, diagnostic and therapeutic indications, and complications of bronchoscopy.

Material and method

This retrospective chart review included children aged between one day and 13 years, admitted to AQWCH, who underwent bronchoscopy (rigid or flexible) procedures between January 2018 and December 2019. All patients were identified by using a computerized search of hospital discharge diagnosis, which was codiﬁed as "pediatric bronchoscopy, flexible, rigid, bronchoalveolar lavage". The main study outcome measure was to evaluate patients' characteristics, diagnostic or therapeutic indications, bronchoalveolar lavage (BAL) analysis, as well as complications of bronchoscopy at AQWCH.

Results

There were 72 pediatric bronchoscopies (rigid and flexible) performed in patients aged less than 13 years old; the reason for bronchoscopy procedure was diagnostic in 51% and both diagnostic and therapeutic in 49%. Cough was the most common symptom (n=53; 74%), and chest recession was the most common clinical finding (n=46; 64%). Foreign body aspiration was the main indication (n=23; 32%), followed by stridor (26%). Consolidation was the most common radiological finding. Foreign body was the common finding, seen in 25% of bronchoscopies, followed by tracheomalacia in 17%. The suspected diagnosis was confirmed in 89%, and management change was needed in 54% of patients. The main complication during the procedure was desaturation (26%), and cough was the main post-bronchoscopy complication (14%). BAL was done for 28 (39%) patients, in which BAL culture was positive in 75%. Rigid bronchoscopy was done when foreign body aspiration was suspected based on positive history in 70%, abnormal physical examination in 60%, and chest X-ray abnormalities in 39% of patients. Sensitivity and specificity for patient history, physical examination, and chest X-ray were 80% and 83%, 66% and 60%, and 40% and 66 %, respectively.

Conclusion

Bronchoscopy is an important tool for evaluating, diagnosing, and treating pediatric respiratory diseases. While it is a safe procedure, it still needs a careful selection of patients as it is invasive.

## Introduction

A bronchoscope is a tool that is used to visualize the upper and lower airways for diagnostic and therapeutic reasons [1,2]. Bronchoscopy is one of the most important diagnostic and therapeutic procedures in pediatric respiratory disease and has advantages over other diagnostic methods. There are two types of pediatric bronchoscopy, flexible (FB) and rigid (RB). The rigid bronchoscope consists of a metal body, and it is inserted under general anesthesia into the tracheobronchial tree. A flexible bronchoscope consists of bundles of optical fibers that are used for imaging and delivering light to the tip. Besides, the bundles are the working channel for intervention and passage of the instrument. It can be done under light sedation or general anesthesia [2].

There are various indications for bronchoscopy: persistent stridor, congenital, anatomical, or acquired anomalies, persistent wheezing, hemoptysis, persistent or recurring atelectasis, persistent or recurring pneumonia, and localized hyperlucency. Other uses of bronchoscopy are bronchoalveolar lavage (BAL), getting biopsy samples, and aspiration of secretions. Examples of therapeutic bronchoscopy are administering medications and removing foreign bodies [1,3-8]. Absolute contraindications for bronchoscopy include severe hypoxemia, hemodynamic instability, and uncorrected hemorrhagic diathesis. Expreterm, severe pulmonary hypertension, and congenital cyanotic cardiomyopathy with increased bronchial collateral circulation are relative contraindications [9]. In addition, instabilities in the cervical spine or atlantooccipital transition are considered relative contraindications for rigid bronchoscopy [10]. The common complication of bronchoscopy is related to anesthesia, mechanical trauma (epistaxis, pneumothorax, and hemoptysis), hypoxemia, laryngospasm, post-lavage fever, and infection [1,11].

In this retrospective chart review (RCR), we reviewed and analyzed data of pediatric patients who had bronchoscopy done between January 1, 2018, to December 31, 2019, during their admission in AQWCH. This study aims to evaluate patients' characteristics, diagnostic and therapeutic indications, and complications of bronchoscopy.

## Materials and methods

Methods and study design

This study is a RCR. We used a timeframe sample and utilized de-identified medical record data of all patients who underwent a bronchoscopy procedure between January 01, 2018, and December 31, 2019. This study was conducted at Al Qassimi Women and Children Hospital (AQWCH), Sharjah, United Arab Emirates (UAE). All selected patients were minors; however, all data were unidentified, anonymous, and stored on password-protected computers accessed by the principal investigator only, and ethical approval was obtained from the Ministry of Health and Prevention Medicine, UAE (Approval number: MOHAP/DXB-REC/JJJ/No.3/2021).

Patient selection

Patients who were hospitalized at AQWCH and had bronchoscopy procedures done between January 1, 2018, and December 31, 2019, were identified retrospectively from the electronic database of the Pediatric Department. We included in this study children between the ages of one day to 13 years who underwent a flexible or rigid bronchoscopy procedure. We excluded repeated bronchoscopy for the same patient in the same admission.

Statistical analysis

Descriptive statistics were used to describe the characteristics of the variables, using frequencies for categorical variables. Data for categorical variables were tested using a Chi-square test and Fisher's exact test. Continuous variables were tested using the T-test and Mann-Whitney U test. Normality was tested using the Shapiro-Wilk test and visualization of histograms. If the continuous variables were not normal, then the Kruskal-Wallis test was used instead. We rounded the percentage up or down to the nearest number of decimals for the purpose of easily understanding without affecting the main result. The alpha value of P-value ≤ 0.05 was used to determine statistical significance.

## Results

The retrospective analysis was done for 72 patients under the age of 13 years who underwent bronchoscopy (FB or RB) between January 2018 and December 2019. Of these patients, 39 were male, and 33 were female, with a median age of 1.06 years (range 0.045-4.05). Regarding the aim of bronchoscopy, 51% was diagnostic, and 49% diagnostic and therapeutic. The signs and symptoms were cough (n=53; 74%), distress (n=47; 65%), failure to thrive (n=32; 44%), fever (n=30; 42%), cyanosis (n=7; 10%), and clubbing (n=6; 8%). Findings on clinical examination were chest recession (n=46; 64%), lung crackles (n=35; 49%), lung`s asymmetric air entry (n=31; 43%), wheezes (n=23; 32%), stridor (n=19; 26%), chest wall malformation (n=6; 8%), and dullness (n=1; 1%) (Table 1).

**Table 1:** Demographic and medical characteristics of patients.

Chracteristics	Frequencies
Gender, n (%)	
Male	39 (54%)
Female	33 (46%)
Age (years) median (IQR)	1.06 (0.045-4.05)
Aim of bronchoscopy, n (%)	
Only diagnostic	37 (51%)
Diagnostic and therapeutic	36 (49%)
Signs and symptoms, n (%)	
Cough	53 (74%)
Distress	47 (65%)
Failure to thrive	32 (44%)
Fever	30 (42%)
Cyanosis	7 (10%)
Clubbing	6 (8%)
Respiratory findings in clinical examination, n (%)	
Recession	46 (64%)
Lung crackles	35 (49%)
Wheezes	23 (32%)
Stridor	19 (26%)
Asymmetric air entry	15 (21%)
Chest wall malformation	6 (8%)
Dullness	1 (1 5%)

Indication for bronchoscopy

The indications of bronchoscopy were foreign body aspiration (n=23; 32%), persistent stridor (n=19; 26 %), BAL (n=15; 21%), persistent pneumonia (n=13; 18%), recurrent pneumonia (n=11; 15%), bronchiectasis (n=10; 12%), interstitial lung disease (n=6; 8%), wheezing (n=5; 7%), difficult extubation (n=7; 10%), chronic cough (n=4; 6%), persistent dyspnea (n=3; 4%), persistent atelectasis (n=2; 3%), difficult intubation (n=2; 3%), hemoptysis (n=1; 1%), pneumonia in an immunocompromised patient (n=1; 1%), unilateral wheezing (n=1; 1%), and hyperlucent lung (n=1; 1%). In the present study, the underlying diseases found were respiratory disorder (n=18; 25%), cardiology disease (n=13; 18%), gastrointestinal disorder (n=8; 11%), and immunodeficiency (n=1; 1%). The decision for bronchoscopy was made by pulmonologists in 70 (97%) patients, and by other specialties in two patients (Table 2).

**Table 2:** Indication, underlying disease, and decision of bronchoscopy

	Frequency, n (%)
Indication for Bronchoscopy	
Foreign body	23 (32%)
Stridor	19 (26%)
Persistent pneumonia	13 (18%)
Bronchoalveolar lavage (BAL)	15 (21%)
Recurrent pneumonia	11 (15%)
Bronchiectasis	10 (12%)
Difficult extubation	7 (10%)
Interstitial lung disease	6 (8%)
Persistent wheezing	5 (7%)
Chronic cough	4 (6%)
Dyspnea	3 (4%)
Difficult intubation	2 (3%)
Persistent atelectasis	2 (3%)
Hemoptysis	1 (1%)
Pneumonia in an immunocompromised patient	1 (1%)
Unilateral wheezing	1 (1%)
Hyperlucent lung	1 (1%)
Underlying disease	
Respiratory disorder	18 (25%)
Cardiology disease	13 (18%)
Neuromuscular disorder	13 (18%)
Gastrointestinal disorder	8 (11%)
Immunodeficiency	1 (1%)
Hematology/oncology disease	1 (1%)
Decision for Bronchoscopy	
Pulmonologist	70 (97%)
Other	2 (3%)

Rigid bronchoscopy

A total of 23 patients underwent RB with suspicion of foreign body aspiration. Of them, 16 (70%) patients presented with a positive history, and in 15 (93%), foreign body was found. Negative history was in seven (30%) patients and in three (42%) of them, foreign body was found. We found abnormal physical examination in 14 (60%) patients, and in 12 (85%) of them, foreign body was extracted. Examination was normal in nine (39%), and foreign body was found in six (67%) patients. Chest X-ray was abnormal in nine (39%), and foreign body was extracted in eight (89%) patients. Chest x-ray was normal in 14 (61%) and foreign bodies were found in 12 (85%) (Table 3). Extracted foreign bodies included 14 organic, four plastic, one sticker, one stone, one candy wrap, one stalk of grape, and one pin. Specificity and sensitivity of patient history, physical examination, and chest X-ray were 80% and 83%, 66% and 60%, and 40% and 66%, respectively (Table 3).

**Table 3:** Sensitivity and specificity of different variables in rigid bronchoscopy

Variable		Total No, n (%)	Foreign Body, n (%)		Specificity, %	Sensitivity, %
			Yes	No		
Patient History	Positive history	16 (70%)	15 (93%)	1 (7%)	80%	83%
	Negative history	7 (30%)	3 (42%)	4 (58%)		
Physical examination	Positive examination	14 (60%)	12 (85%)	2 (15%)	66%	60%
	Normal examination	9 (40%)	6 (67%)	3 (33%)		
Chest X-ray	Abnormal Chest X-ray	9 (39%)	8 (89%)	1 (11%)	40%	66%
	Normal Chest X-ray	14 (61%)	12 (85%)	2 (15%)		

Radiologic findings

Radiological findings were tested at the time of admission. Chest X-ray study for all patients (n=72) showed consolidation in 29 (40%), pneumonia in 21 (29%), normal study in 18 (25%), unilateral hyperinflation in 11 (15%), atelectasis in nine (13%), pleural effusion in seven (10%), localized air trapping in three (4%), pneumothorax in one (1%), mediastinal shift in one (1%), mediastinal mass in one (1%), and visible foreign body in one (1%). Chest CT was done for 40 (56%) patients and it revealed consolidation in 29 (73%), bronchiectasis in 12 (31%), ground glass opacity in 12 (30%), pleural effusion in eight (20%), normal findings were in three (8%) unilateral hyperinflations in three (8%), septation in three (8%), cystic malformation in one (3%), and visible foreign body in one (3%) (Table 4).

**Table 4:** Radiological findings

Radiologic Findings on Admission	Frequencies, n (%)
Consolidation on the chest X-ray study	29 (40%)
Pneumonia on the chest X-ray study	21 (29%)
Normal radiograph on the chest X-ray study	18 (25%)
Unilateral hyperinflation on the chest X-ray study	11 (15%)
Atelectasis on the chest X-ray study	9 (13%)
Pleural effusion on the chest X-ray study	7 (10%)
Localized air trapping on the chest X-ray study	3 (4%)
Mediastinal shift on the chest X-ray study	1 (1%)
Mass on the chest X-ray study	1 (1%)
Visible foreign body on the chest X-ray study	1 (1%)
Pneumothorax on the chest X-ray study	1 (1%)
Chest CT study done	40 (56%)
Consolidation on the chest CT study	29 (73%)
Bronchiectasis on the chest CT study	12 (31%)
Ground glass opacity findings on the chest CT study	12 (30%)
Pleural effusion on the chest CT study	8 (20%)
Unilateral hyperinflation on the chest CT study	3 (8%)
Septation on the chest CT study	3 (8%)
Normal findings on the chest CT study	3 (8%)
Visible foreign body findings on the chest CT study, no (%)	1 (3%)
Cyst on the chest CT study, no (%)	1 (3%)

Bronchoscopy findings

The bronchoscopy procedure result showed foreign body in 18 (25%), tracheomalacia in 12 (17%), normal findings in 11 (15%), airway narrowing in eight (11%), granulation tissue in six (8%), inflammation in seven (10%), vocal cord swelling in seven (10%), mucus plug was seven (10%), airway compression in seven (10%), subglottic stenosis in six (8%), laryngomalacia in three (4%), glottic stenosis in two (3%), hemangioma in two (3%), bleeding in one (1%), airway malformation in one (1%), bronchomalacia in one (1%), and vocal cord immobility in one (1%). The suspected diagnosis was confirmed in 64 (89%), management was changed in 39 (54%), and the diagnosis was changed in 34 (47%) (Table 5).

**Table 5:** Bronchoscopy findings and efficacy

Bronchoscopy Findings	Frequencies, n (%)
Foreign body	18 (25%)
Tracheomalacia	12 (17%)
Normal bronchoscopy	11 (15%)
Airway narrowing	8 (11%)
Airway compression	7 (10%)
Inflammation	7 (10%)
Mucus plug	7 (10%)
Vocal cord swelling	7 (10%)
Granulation tissue	6 (8%)
Subglottic stenosis	6 (8%)
Laryngomalacia	3 (4%)
Hemangioma	2 (3%)
Glottis stenosis	2 (3%)
Vocal cord immobility	1 (1%)
Bleeding	1 (1%)
Airway malformation	1 (1%)
Bronchomalacia	1 (1%)
Bronchoscopy efficacy	
Suspected diagnosis confirmed	64 (89%)
Change of management	39 (54%)
Change of diagnosis after the procedure	34 (47%)

Complications during and after the bronchoscopy procedure

Complications found during the bronchoscopy included desaturation in 19 (26%), fever in eight (11%), bleeding in two (3%), laryngeal spasm in two (3%), and bronchial spasm in one (1%). Complications post bronchoscopy included cough in 10 (14%), bleeding in three (4%), desaturation in one (1%), laryngeal spasm in one (1%), bronchial spasm in one (1%), and vomiting in one (1%) (Table 6).

**Table 6:** Complications seen during and after bronchoscopy

Complications	Freuencies, n (%)
During the Bronchoscopy Procedure	
Desaturation	19 (26%)
Bleeding	2 (3%)
Laryngeal spasm	2 (3%)
Apnea	1 (1%)
Bronchial spasm	1 (1%)
After the Bronchoscopy Procedure	
Cough	10 (14%)
Fever	8 (11%)
Bleeding	3 (4%)
Laryngeal spasm	1 (1%)
Desaturation	1 (1%)
Bronchial spasm	1 (1%)
Vomiting	1 (1%)

BAL

**Figure 1:**
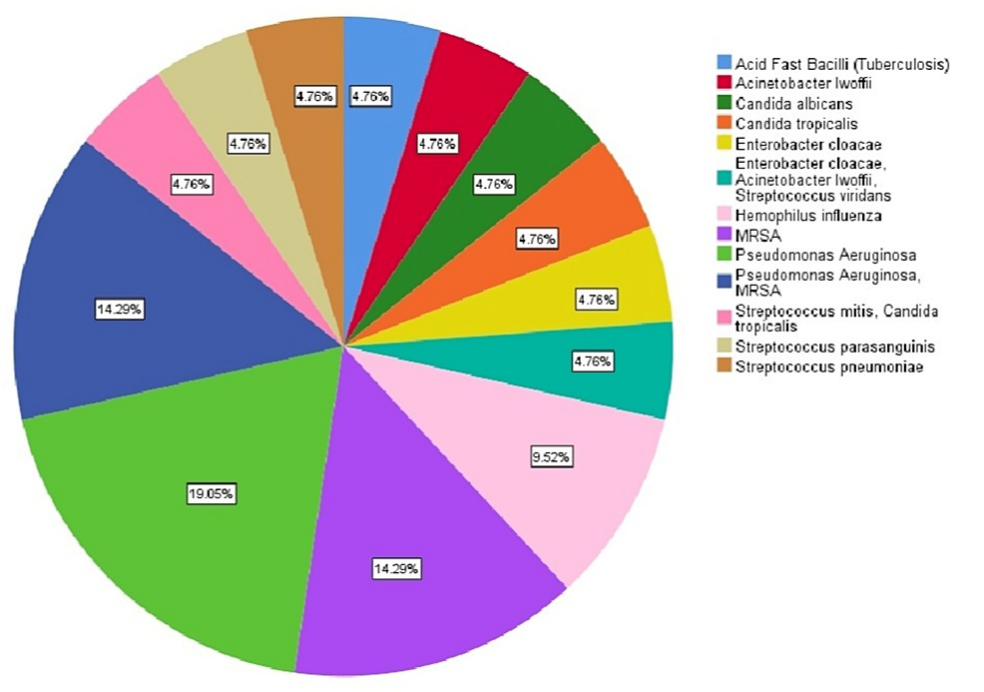
The cultured pathogen MRSA: methicillin-resistant *Staphylococcus aureus *

BAL was done for a total of 28 (39%) patients. The diagnosis was changed after the BAL result in 22 (79%), and the pathogen was detected by BAL in 21 (75%) with a negative culture in seven (25%) (Table 7). The common organism was *Pseudomonas aeruginosa* (Figure 1).

**Table 7:** Bronchoalveolar lavage result BAL: bronchoalveolar lavage

	Frequencies, n (%)
BAL	28 (39%)
Diagnosis changed after BAL result	22 (79%)
Pathogen detected by BAL	21 (75%)

## Discussion

This study aimed to reveal the most important indications, complications, and diagnostic and therapeutic yield of pediatric bronchoscopy. To our knowledge, this is the first study describing indications and highlighting possible complications of bronchoscopy procedures among the pediatric population in the UAE. The macroscopic (static and dynamic) and microscopic (cultures, histocytology) givens, collected during the bronchoscopy are important information allowing ruling in or out the suspected diagnosis; in addition, revealing some unpredicted findings can lead to the final diagnosis.

Of the total of 72 bronchoscopies, RB was 23 (32%), and 49 (68%) was FB. The most common symptom was cough at 74%, and the most common presenting clinical finding was lung crackles at 49%. Persistent wet cough for four weeks and more should be investigated via bronchoscopy [12,13], and in our study, only 6% of patients underwent bronchoscopy due to wet cough.

In this study, foreign body aspiration was the commonest indication for RB (32%), followed by stridor (26%) for FB. In a multi-center survey study done in Europe, foreign body aspiration was seen in 20 of 30 patients. There are papers that reported the extraction of foreign body with flexible bronchoscopy [14]. Despite these papers, as mentioned in the Greek study [15], we believe the best tool for extraction is rigid bronchoscopy. Similar to other studies, stridor was the most common indication for flexible bronchoscopy [16], but contrary to the other studies, the most common bronchoscopic finding was tracheomalacia. A foreign body was found in only 25% of all bronchoscopies and 78% of RB. Similar to other studies, chest X-rays were normal in 47% of patients who were suspected of having a foreign body, which can reach up to 66% [17-21].

We tested the outcome of bronchoscopy, which was slightly higher compared to international studies, and was valuable for diagnosis in 89% [14,22-25]. Both types of bronchoscopy are safe procedures. The common complications that are seen are desaturation, hypoxemia, cough and bronchospasm, trauma, and obstruction of the airway due to edema, hemorrhage, pneumothorax, fever, and infections [26,1].

In our study, the complications that occurred during and post bronchoscopy were considered to be minor, and desaturation was the common complication during the bronchoscopy. At the same time, cough was the most common complication post bronchoscopy. According to Rosenthal, fever is one of the most common consequences of bronchoscopy, especially post BAL [11]. In the present study, however, there was no fever related to bronchoscopy either due to underlying disease (infection) or due to early discharge of the patient post procedure.

In the present study, the diagnostic yield of BAL was high (79%), and BAL culture was positive in 75%. Earlier studies have revealed the diagnostic yield of BAL between 33-80% [27-30]. *Pseudomonas* was the comment organism found in the culture, and this was due to the nature of patients who underwent bronchoscopy procedures.

This study has a number of limitations, including small sample size and retrospective design. This might have affected some of our results, particularly those related to cell count and differentiation (histocytology) in BAL and the risk factors for desaturation.

## Conclusions

Bronchoscopy is one of the most important tools in pediatric pulmonology. The most common indication for bronchoscopy in children is stridor and foreign body aspiration in our study. Bronchoscopy is a diagnostic tool not only for airway anatomy but also to obtain BAL for cell analysis and culture, which can differentiate infection from inflammation and diagnose diseases. While it is a safe procedure and complications are rare, especially with expert bronchoscopists, life-threatening complications are still possible. It requires different preparation with each indication of the procedure to avoid such complications. In addition, bronchoscopy is an important tool for interventions such as bronchial and lung biopsy, intubation of children in some situations, and balloon bronchial dilatation with stents. The type of bronchoscopy (RB or FB) depends on the indications.

## References

[1] Midulla F, de Blic J, Barbato A (2003). Flexible endoscopy of paediatric airways. Eur Respir J.

[2] Sharma S, Sawant P (2015). Pulmonary aspiration and management with immediate rigid bronchoscopy. Ped Anesth Crit Care J.

[3] Kabir AL, Majumder JU, Mridha AA, Rahman M, Amin MR (2005). Pediatric ﬂexible ﬁberoptic bronchoscopy. Bangladesh J Child Health.

[4] de Blic J, Marchac V, Scheinmann P (2002). Complications of flexible bronchoscopy in children: prospective study of 1,328 procedures. Eur Respir J.

[5] Nussbaum E (2002). Pediatric fiberoptic bronchoscopy: clinical experience with 2,836 bronchoscopies. Pediatr Crit Care Med.

[6] Peng YY, Soong WJ, Lee YS, Tsao PC, Yang CF, Jeng MJ (2011). Flexible bronchoscopy as a valuable diagnostic and therapeutic tool in pediatric intensive care patients: a report on 5 years of experience. Pediatr Pulmonol.

[7] Puhakka H, Kero P, Erkinjuntti M (1987). Pediatric bronchoscopy during a 17-year period. Int J Pediatr Otorhinolaryngol.

[8] Faro A, Wood RE, Schechter MS (2015). Official American Thoracic Society technical standards: flexible airway endoscopy in children. Am J Respir Crit Care Med.

[9] Brownlee KG, Crabbe DC (1997). Paediatric bronchoscopy. Arch Dis Child.

[10] Schramm D, Freitag N, Nicolai T (2021). Pediatric airway endoscopy: recommendations of the Society for Pediatric Pneumology. Respiration.

[11] Rosenthal M (2003). Bronchoscopy and infection. Paediatr Respir Rev.

[12] Morice AH, Millqvist E, Bieksiene K (2020). ERS guidelines on the diagnosis and treatment of chronic cough in adults and children. Eur Respir J.

[13] Kantar A, Chang AB, Shields MD (2017). ERS statement on protracted bacterial bronchitis in children. Eur Respir J.

[14] Godfrey S, Avital A, Maayan C, Rotschild M, Springer C (1997). Yield from flexible bronchoscopy in children. Pediatr Pulmonol.

[15] Ramírez-Figueroa JL, Gochicoa-Rangel LG, Ramírez-San Juan DH, Vargas MH (2005). Foreign body removal by flexible fiberoptic bronchoscopy in infants and children. Pediatr Pulmonol.

[16] Swanson KL, Prakash UB, Midthun DE, Edell ES, Utz JP, McDougall JC, Brutinel WM (2002). Flexible bronchoscopic management of airway foreign bodies in children. Chest.

[17] Figuerola Mulet J, Osona Rodríguez de Torres B, Llull Ferretjans M, Román Piñana JM (2005). Contribution of flexible bronchoscopy to the diagnosis of upper airway alterations (Article in Spanish). An Pediatr (Barc).

[18] Ciftci AO, Bingöl-Koloğlu M, Senocak ME, Tanyel FC, Büyükpamukçu N (2003). Bronchoscopy for evaluation of foreign body aspiration in children. J Pediatr Surg.

[19] Mohammad M, Saleem M, Mahseeri M (2017). Foreign body aspiration in children: a study of children who lived or died following aspiration. Int J Pediatr Otorhinolaryngol.

[20] Zaytoun GM, Rouadi PW, Baki DH (2000). Endoscopic management of foreign bodies in the tracheobronchial tree: predictive factors for complications. Otolaryngol Head Neck Surg.

[21] Cavel O, Bergeron M, Garel L, Arcand P, Froehlich P (2012). Questioning the legitimacy of rigid bronchoscopy as a tool for establishing the diagnosis of a bronchial foreign body. Int J Pediatr Otorhinolaryngol.

[22] Cataneo AJ, Cataneo DC, Ruiz RL Jr (2008). Management of tracheobronchial foreign body in children. Pediatr Surg Int.

[23] Maffey AF, Berlinski A, Schkair JC, Teper AM (2008). Flexible bronchoscopy in a pediatric pulmonology service (Article in Spanish). Arch Argent Pediatr.

[24] Wood RE (1985). The diagnostic effectiveness of the flexible bronchoscope in children. Pediatr Pulmonol.

[25] Raine J, Warner JO (1991). Fibreoptic bronchoscopy without general anaesthetic. Arch Dis Child.

[26] Kirvassilis F, Gidaris D, Ventouri M (2011). Flexible fiberoptic bronchoscopy in Greek children. Hippokratia.

[27] Kottmann RM, Kelly J, Lyda E (2011). Bronchoscopy with bronchoalveolar lavage: determinants of yield and impact on management in immunosuppressed patients. Thorax.

[28] Hummel M, Rudert S, Hof H, Hehlmann R, Buchheidt D (2008). Diagnostic yield of bronchoscopy with bronchoalveolar lavage in febrile patients with hematologic malignancies and pulmonary infiltrates. Ann Hematol.

[29] Cordonnier C, Escudier E, Verra F, Brochard L, Bernaudin JF, Fleury-Feith J (1994). Bronchoalveolar lavage during neutropenic episodes: diagnostic yield and cellular pattern. Eur Respir J.

[30] Todd T, Enoch DA (2009). Role of bronchoalveolar lavage in evaluating new pulmonary infiltrates on computed tomography in haematology patients with fever unresponsive to broad-spectrum antibiotics. J Med Microbiol.

